# Oral cholera vaccine coverage evaluation survey: Forcibly Displaced Myanmar Nationals and host community in Cox's Bazar, Bangladesh

**DOI:** 10.3389/fpubh.2023.1147563

**Published:** 2023-07-05

**Authors:** Md. Omar Qayum, Mallick Masum Billah, Mohammad Ferdous Rahman Sarker, A. S. M. Alamgir, Mehejabin Nurunnahar, Manjur Hossain Khan, M. Salim Uzzaman, Alden Henderson, Tahmina Shirin, Meerjady Sabrina Flora

**Affiliations:** ^1^Institute of Epidemiology, Disease Control and Research (IEDCR), Dhaka, Bangladesh; ^2^Centers for Disease Control and Prevention (CDC), Atlanta, GA, United States; ^3^Directorate General of Health Services (DGHS), Dhaka, Bangladesh

**Keywords:** cholera, oral cholera vaccine, vaccine coverage, vaccination, Forcibly Displaced Myanmar Nationals, host community, refugee camps, Bangladesh

## Abstract

**Introduction:**

Cholera remains a significant public health concern in many parts of the world, particularly in areas with poor sanitation and hygiene. Bangladesh and other impoverished nations have been severely affected by cholera outbreaks, especially in areas with a high population density. In order to mitigate the spread of cholera, oral cholera vaccines (OCVs) are recommended as a prophylactic measure. In May 2018, 775,666 of the Forcibly Displaced Myanmar Nationals (FDMN) in the registered and makeshift camps and 103,605 of the residents in the host community received two doses of OCV Shanchol^TM^ in Cox's Bazar, Bangladesh, because the conditions in the area favored the transmission of cholera and other waterborne diseases. This study aimed to assess the coverage of OCV among the FDMN and the host community in Cox's Bazar.

**Methods:**

In August 2018, we enrolled 4,240 respondents for this study following the “World Health Organization (WHO) Vaccination Coverage Cluster Surveys: Reference Manual (2018).” The coverage survey was conducted with three strata of the population: the host community from the Teknaf Upazila, the registered camp, and the makeshift camp from the Ukhia Upazila. We collected information regarding OCV coverage, demographic characteristics, and knowledge and behaviors of people toward the vaccine. The data were analyzed using descriptive statistics.

**Results:**

According to our study, the overall OCV coverage was 85%, with 68% in the host community, 91% in the registered camp, and 98% in the makeshift camp. The lower coverage in the host community was due to residents unaware of the vaccination campaign, the unavailability of vaccines, and unaware where to go for vaccination.

**Discussion:**

Our findings demonstrate that the OCV campaign in the FDMN camps was successful, reaching over 90% coverage, while coverage in the host community was much lower. In order to make sure that OCV vaccination efforts are reaching the target population and having the desired impact, our study emphasizes the need to inform the target population of when and where to get vaccinated.

## Introduction

Cholera is an acute diarrheal disease, which is caused by infection due to toxigenic strains of the bacterium *Vibrio cholerae*. Cholera remains a significant public health concern globally, with an estimated 1.3 to 4 million cases and 21,000 to 143,000 deaths reported annually (1). In Bangladesh, where cholera is endemic, the risk of outbreaks is particularly high in areas with poor sanitation and limited access to clean water, such as in the Rohingya refugee camps in Cox's Bazar district, which are particularly vulnerable to epidemics. Every year, there are at least 100,000 cases and ~4,500 fatalities in Bangladesh alone (1). Bangladesh remains endemic for cholera with a biannual peak in certain areas of the country (2). Despite the fact that cholera can affect people of different ages, the majority of fatal cases are reported in young children. Although adults are also in danger, children under the age of 5 suffer the majority of the burden of the disease (3, 4).

In 2014, the Global Task Force on Cholera Control (GTFCC), a World Health Organization (WHO)-coordinated network of partners, began working with several countries on national cholera control plans. The GTFCC's roadmap aims to reduce cholera mortality by 90% and eliminate local transmission in at least 20 countries by 2030 by providing vaccines and improved water, sanitation, and hygiene (WASH) facilities (5). The oral cholera vaccine (OCV) stockpile was established by the WHO for deployment during emergencies, and the vaccine has been available in Bangladesh's high-risk districts since 2013, reducing the likelihood of cholera outbreaks (6, 7).

In late August 2017, more than 650,000 Forcibly Displaced Myanmar Nationals (FDMNs) from the northern parts of Myanmar's Rakhine State began arriving in the Cox's Bazar, a coastal area in the southern part of Bangladesh, joining an estimated 300,000 FDMNs who had already arrived during prior waves of relocation (8). The Needs and Population Monitoring (NPM) program revealed that as of 25 May 2018, there were supposedly 915,000 FDMNs living in Cox's Bazar. Of these, 702,000 had just arrived as of 25 August 2017 (9). Most of these individuals sought refuge in Teknaf and Ukhia Upazilas in Cox's Bazar district and Naikhongchhari Upazila in Bandarban district, which were made up of temporary settlements, refugee camps, and host communities. Another significant surge of FDMNs arrived in October 2016, with the majority living in Cox's Bazar Kutupalong Makeshift Settlement (KMS) and the Balukhali Makeshift Settlement (BMS). In July 2017, the population of the BMS was projected to be 20,000 (10). The KMS and BMS have reportedly received 439,600 refugees since August 2017 (11). Due to this, the pre-existing makeshift settlements experienced quick and significant expansions, which ultimately led to the consolidation of the two makeshift settlements into a single, sizable expansion site. Since there was no infrastructure in place when the refugees arrived, the living conditions in the extensions were worse than those in the Makeshift Settlements.

The overcrowding of people has put pressure on the basic healthcare infrastructure and services along with already poor access to safe drinking water, proper hygiene, and sanitation facilities available. In total, 60% of the total incoming FDMN population were young children, who were vulnerable to developing vaccine-preventable diseases. In Cox's Bazar, there were a large number of cases of acute watery diarrhea (36,533) from January to July 2018, according to the Mortality and Morbidity Weekly Bulletin published by the US Centers for Disease Control and Prevention (CDC) on 20 February 2018 (12). Therefore, to stop and manage the spread of cholera and other waterborne diseases, it is essential to provide safe water and sanitation as well as OCVs to the refugees and host communities for reducing the outbreak. As a preventative measure to lower the threat of cholera outbreaks in the refugee camps, the Government of Bangladesh (GoB) requested the International Coordinating Group (ICG) to allocate 900,000 doses of OCVs from the global stockpile (13). The GoB, acting as the lead with technical support from the International Center for Diarrhoeal Disease Research, Bangladesh (icddr,b), other international agencies [United Nations International Children's Emergency Fund (UNICEF), WHO, Médecins Sans Frontières (MSF), International Organization for Migration (IOM)], and national non-governmental organizations (NGOs) under the larger platform of the health sector, undertook an immediate massive OCV campaign. From 6 to 13 May 2018, the first and second doses of the second round OCV campaign were conducted throughout the camps, and OCV (Shanchol^TM^) was administered to a total of 879,273 individuals, who were 1 year of age or older. Among them, 775,666 were from the FDMN and 103,605 were from the host community (14). However, the coverage of OCV among FDMNs and the host community in Cox's Bazar remains unknown. This study was conducted to evaluate the coverage of OCVs for the first time among both the populations of the FDMNs and the host community in Cox's Bazar following the OCV campaign. This is the first-ever evaluation study from Bangladesh government sites where the host community and camp people both were enrolled for this maiden study. The findings of this study will offer important details about the coverage of OCV in these groups and highlight places where vaccination campaigns may be strengthened and will have important policy implications for the GoB and international organizations, who are working together to prevent cholera outbreaks in Cox's Bazar. Moreover, the results will contribute to a better understanding of the prevention and control of cholera and improve knowledge, attitudes, and practices among these people, resulting in more successful public health interventions.

## Materials and methods

### Study period, design, and population

We conducted a cross-sectional survey using a multistage cluster sampling design among FDMNs in the registered and makeshift camps in Ukhia Upazila and in the host community of Teknaf Upazila in Cox's Bazar who were targeted to receive OCVs from 6 to 13 May 2018. Any individual who met the following criteria was eligible to participate in the survey:

1) 1 year of age or older at the time of the first round of vaccination,
2) residents of selected households at the time of the OCV campaign in the communities targeted for OCV vaccination, and
3) if written informed consent is given by the participant for the survey (or by a responsible adult member of the household if the participant is younger than 15 years).

The exclusion criteria for this study included people living in areas where the campaign was not implemented, pregnant women, and children younger than 1 year.

The study had three population strata: the registered camp, makeshift camp, and host community. Each stratum had a unique location that differed by access to healthcare, community sanitation, population density, and access to potable water. Given these differences, we felt that these differences will affect OCV coverage.

### Working definitions

#### Kutupalong registered camp (KRC)

Although KRC closed registration in 1992, refugees continued to arrive and settle around the official camp and formed the Kutupalong Makeshift Settlement (KMS). The KRC and KMS make up one of Bangladesh's largest Rohingya refugee settlements. The KRC has a population of ~32,000 and is one of the only two camps containing registered refugees (15).

#### Makeshift camp

It applies to temporary shelters. The majority of the refugees in Cox's Bazar district live in pre-existing camps, settlements, settlement extensions (additions to pre-existing settlements), spontaneous settlements, newly formed settlements with little support, and among the host community. According to reports, the majority of new refugees (578,000 individuals) were residing in makeshift or new spontaneous settlements, while 46,000 were staying in host communities (16).

#### Host community

Local population who are Bangladeshi citizens residing before and after the Rohingya influx are considered host communities. In the context of this survey, the host community may encompass the camp, or may simply neighbor the camp but interact with, or otherwise be impacted by, the refugees residing in the camp. The population of the host community is 336,000.

### Sample size

The sample size was calculated using the *World Health Organization (WHO) Vaccination Coverage Cluster Surveys: Reference Manual* (17). The population was divided into three strata: the host community of Teknaf Upazila (population 152,000), the registered camp (population 256,000), and the makeshift camp for Ukhia Upazila (population 597,000). For each age group, the sample size was 432 based on an expected 90% OCV coverage and a design effect of 2. This sample size was then multiplied by three to accommodate each stratum. As a result, 432 respondents were enrolled from each age group which resulted in a total of 1,296 respondents in each stratum. The expected number of clusters per stratum was 62, and the total number of households to visit per cluster was 7 for achieving the desired sample size according to the cluster survey manual (17).

### Sampling procedure

#### Makeshift camps

The Majhi, who acted as local leaders of FDMNs, provided a list of 1,076 clusters, which included all the households in the camp. In total, 63 were randomly selected for the survey. Each cluster has about 20 households. Upon arriving at the cluster, we listed each household and randomly selected one household as the starting point. The Majhi helped us in finding the starting household. Within each household, we recruited one person in the following age groups: 1–4, 5–14, and ≥15 years. If the household had more than one person in the same age group, one person was randomly selected. If there was no eligible person, we proceeded to the nearest house on the right when exited the door of the starting house.

Upon completing an interview and exiting the house, we approached the house to the right to continue our random walk to recruit households for the survey. The recruitment of study participants continued until there were at least 432 participants in each stratum and age group.

#### Registered camps (RC)

The team collected demographic and relevant information from the local office of The Refugee Relief and Repatriation Commissioner (RRRC), The United Nations High Commissioner for Refugees (UNHCR), and the office of the Upazila Nirbahi Officer (UNO) as Bangladesh Administrative Service. There were 453 sheds in the RC according to the mentioned authorities. From the 453 sheds, we randomly selected 148 sheds assuming that each shed has 10 households with a family size of 8 people and added 22 sheds for refusals or people who are not at home. Every two sheds were considered a cluster. From each cluster, we randomly selected one household for a starting point with the help of the camp committee members. The selection of households and participants was identical to the makeshift camp described above.

#### Host community

There are six unions in Teknaf Upazila, Cox's Bazar. The Saint Martin union was not targeted for the OCV campaign and was excluded from the survey. According to the GoB Expanded Programme on Immunization (EPI) micro plan of Teknaf Upazila Health Complex, each union has three wards and each ward was divided into eight sub-blocks. In total, 80 sub-blocks of five unions were included for the OCV coverage. Each block was considered a cluster. From 80 sub-blocks, we randomly selected 63 clusters. From each cluster, we randomly selected one household for a starting point with the help of health assistants, family welfare assistants, and community healthcare providers. The selection of households and participants was identical to the makeshift camp described above.

#### Enrollment process

From the randomly selected clusters, we collected data from selected households (HH). Each HH was visited up to three different times on the same day in an attempt to enroll in the survey. Mothers or caretakers of eligible children aged 1–14 years were interviewed; if he/she was unavailable, another adult member of the family was interviewed. Persons aged 15 years and older were directly interviewed about their vaccination status. The rationale for categorizing the population into three age groups (1–4, 5–14, and ≥15 years) is based on the information from previous studies, which showed that age is an important factor for predicting the risk of cholera infection and severity of sickness (3, 4, 18). Children under 5 years are more susceptible to cholera infection due to their underdeveloped immune systems, while older children and adults are more likely to become infected with cholera through either the consumption of contaminated food and water or fecal–oral spread.

We chose study participants from both the FDMN and the host community in Cox's Bazar using a stratified random sampling technique. To minimize the sampling bias, the sample was stratified by location and age. To minimize further selection bias, we confirmed that all eligible participants within each selected HH had an equal chance of being included in the study.

### Data collection and analysis

Data were collected by interview teams using a standardized questionnaire on handheld devices. The survey tool was pre-tested to ensure that the questions were understandable to interviewees in terms of translation accuracy, question comprehension, and appropriate response categories. The questionnaire was revised from findings during the pre-test prior to conducting the survey. Data were collected verbally during interviews, although vaccination cards were checked if available. To ensure the quality of the data collected, the interview team followed the standardized procedures for data collection, management, and analysis. All the interviewers received training on the vaccine, the vaccination campaign, the questionnaire, and the data collection techniques. The study field supervisors routinely monitored and supervised the accuracy and comprehensiveness of the information gathered by the interviewers. The data were transferred daily to the IEDCR server. A dashboard was formulated for day-to-day checking and updating data in the central server and maintaining the quality and validity of data. Descriptive statistics were used to evaluate the vaccination coverage survey. All the statistical analysis in this study was performed using Stata version 14 (College Station, TX, USA).

### Ethical implications

Verbal consent was obtained prior to initiating the OCV coverage survey questionnaire. Consent was obtained by reading a script to all respondents or caregivers who were asked to participate in the survey. Interviews were conducted with those who gave verbal informed consent and documented on paper forms. Reasons for non-response/refusal were documented. Non-participating households were not replaced. Household names were collected for monitoring and accountability of interviewer teams, and the collection of identifier data could result in a loss of confidentiality for participants. The data were kept in a password-protected database to maintain security. This study was reviewed and approved by the Institutional Review Board of the Institute of Epidemiology, Disease Control and Research (IEDCR), Bangladesh.

## Results

We enrolled a total of 10,041 members from the targeted households of the three study groups, namely the makeshift camp, registered camp, and host community groups. Table 1 represents the number and proportion of household members in each age group (1–4, 5–14, and ≥15 years) in the three study groups. Overall, the data show that the population in the makeshift camps tended to be younger with a high proportion of children in the age groups 1–4 and 5–14 years compared to those in the registered camps and host communities (Table 1).

**Table 1:** Age distribution of respondents' household members by age group in three study groups (*N* = 10,041).

**Age group (years)**	**Study group**		
	**Makeshift camp (non-registered) % (** ***n*** **/** ***N*** **)**	**Registered camp % (** ***n*** **/** ***N*** **)**	**Host community % (** ***n*** **/** ***N*** **)**
1–4	39.7 (803/2,021)	29.9 (606/2,021)	30.2 (612/2,021)
5–14	35.9 (1,136/3,165)	33.8 (1,071/3,165)	31.3 (958/3,165)
≥15	26.1 (1,266/4,855)	35.9 (1,746/4,855)	37.9 (1,843/4,855)

There was a total of 85% OCV coverage including 68% in the host community, 91% in the registered camp, and 98% in the makeshift camp (Table 2). The table shows that the age group of individuals aged ≥15 years had the lowest coverage across all sites when considering age and sex distribution. Specifically, only 82.5% of respondents in the ≥15 age group had received their vaccinations. However, the 5–14 age group had the highest coverage rate among all three study groups.

**Table 2:** Estimated OCV coverage by vaccinee's age, sex, and strata (*N* = 4,240).

**Characteristics**	**Total (*N*)**	**Vaccination status**
		**Yes**, ***n*** **(%)**
**Age group (years)**		
**Makeshift camp**		
1–4	444	442 (99.5)
5–14	443	440 (99.3)
≥15	483	457 (94.6)
Total	1,370	1,339 (97.7)
**Registered camp**		
1–4	443	419 (94.6)
5–14	468	450 (96.2)
≥15	536	442 (82.5)
Total	1,447	1,311 (90.6)
**Host community**		
1–4	454	349 (76.8)
5–14	464	379 (81.6)
≥15	505	241 (47.7)
Total	1,423	969 (68.1)
**Sex**		
**Makeshift camp**		
Male	661	645 (97.6)
Female	709	694 (97.9)
Total	1,370	1,339 (97.7)
**Registered camp**		
Male	560	516 (92.1)
Female	887	795 (89.6)
Total	1,447	1,311 (90.6)
**Host community**		
Male	569	408 (71.7)
Female	854	561 (65.7)
Total	1,423	969 (68.1)

In comparison to the makeshift and registered camps, the vaccination coverage of the host community was lower in terms of age group (Table 2). In the host community, children aged 5 to 14 years had the highest coverage of vaccinations (81.6%), while children equal or older than 15 years had the lowest coverage (47.7%). In the makeshift camp, there was a high level of vaccination (98%), and only very few respondents were not immunized.

When considering sex, we observed that the coverage was generally higher among male compared to female participants, except in the makeshift camp (Table 2). There was no difference in vaccination rates among the sex except in host communities, where female participants were less likely to get vaccinated than male participants.

We have also collected data on OCV coverage among the different age groups considering both vaccination cards and recall information (Table 3). In terms of recalled information, the registered camp had a higher proportion of individuals compared to those with vaccination cards. However, the host community and makeshift camp had relatively lower proportions of individuals recalled to have received the vaccine compared to the registered camp. Overall, 29.4% of vaccinated respondents showed their vaccine card, whereas 70.6% of respondents recalled. Specifically, among the study groups, 41.2% of respondents from the makeshift camp, 21.7% of the registered camp, and 25.4% of the host community respondents were able to show their vaccine cards.

**Table 3:** Status of OCV coverage based on vaccination cards and recalls.

**Age group (years)**	**Vaccine card (%)**	**Recall (%)**	**Total**
**Makeshift camp**			
1–4	204 (45.9)	238 (53.6)	442
5–14	206 (46.5)	234 (52.8)	440
≥15	142 (29.4)	315 (65.2)	457
Total	552 (41.2)	787 (58.8)	1,339
**Registered camp**			
1–4	121 (27.3)	298 (67.3)	419
5–14	119 (25.4)	331 (70.7)	450
≥15	45 (8.4)	397 (74.1)	442
Total	285 (21.7)	1,026 (78.3)	1,311
**Host community**			
1–4	107 (23.6)	242 (53.3)	349
5–14	109 (23.5)	270 (58.2)	379
≥15	30 (5.9)	211 (41.8)	241
Total	246 (25.4)	723 (74.6)	969

We have also investigated the probable reasons for non-vaccination among the three sites. The most common reasons for not being vaccinated were lack of awareness (individuals did not know about the campaign and not knowing the place and date). Specifically, among the unvaccinated participants in the makeshift camp, 15 individuals (15.8%) mentioned that they did not participate in the campaign, 30 individuals (31.6%) reported being too busy, and 21 individuals (22.1%) stated that they were unaware of the vaccination. These were the most commonly cited reasons provided by campers who did not receive the immunization (Table 4A).

**Table 4A:** Most frequent reasons for not taking vaccine in the makeshift camp.

**Reason**	**Frequency**	**Percentage**
Busy/no time	30	31.6
Did not know about vaccination activities	21	22.1
Was absent during the campaign	15	15.8
Forgot to go	10	10.5
Fear of vaccine/do not think vaccines are safe/vaccines can cause harm	7	7.4
Did not know when or where to go	5	5.3
The bad taste of the vaccine	3	3.2
The person was sick during the campaign	2	2.1
Long waiting time	1	1.1

Among the unvaccinated individuals in the registered camps, the primary reasons cited for non-immunization were uncertainty regarding vaccination (51 individuals, 17.3%), lack of attendance during vaccination (49 individuals, 16.7%), and the belief that vaccination was unnecessary (49 individuals, 16.7%). These were identified as the top three reasons reported by participants who did not receive the vaccine (Table 4B).

**Table 4B:** Most frequent reasons for not taking vaccine in the registered camp.

**Reason**	**Frequency**	**Percentage**
Did not know about vaccination activities	51	17.3
Was absent during the campaign	49	16.7
Did not think vaccination was necessary/important	49	16.7
The bad taste of the vaccine	32	10.9
Vaccinator refused to vaccinate	24	8.2
Did not know when or where to go	21	7.1
Fear of vaccine/did not think vaccines are safe/vaccines can cause harm	19	6.5
The person was sick during the campaign	16	5.4
Fear of side effects	13	4.4
Clinic was closed	6	2.0

We have found a vaccination coverage gap in the host community compared with the makeshift camp and the registered camp. Among those who were not immunized in the host community, 304 individuals (41.5%) reported being unaware of vaccination programs, 79 individuals (10.8%) stated that the vaccine was unavailable, and 73 individuals (10%) expressed uncertainty regarding where to receive an immunization (Table 4C).

**Table 4C:** Most frequent reasons for not taking vaccine in the host community.

**Reason**	**Frequency**	**Percentage**
Did not know about vaccination activities	304	41.5
Vaccines are not available	79	10.8
Did not know when or where to go	73	10.0
Vaccinator was not there	53	7.2
Did not think vaccination was necessary/important	47	6.4
Was absent during the campaign	46	6.3
Vaccinator refused to vaccinate	26	3.5
Forgot to go	24	3.3
The bad taste of the vaccine	17	2.3

## Discussion

We have conducted an OCV coverage survey of FDMN and the host community in Cox's Bazar, Bangladesh, which provides valuable insights into the vaccination coverage among this population. In our study, we found that the total OCV coverage was 85% with 68% in the host community, 91% in the registered camp, and 98% in the makeshift camp. A recent study regarding the coverage survey of OCV and other vaccines for preventable diseases in FDMNs showed results that are almost consistent with our study, where the overall coverage of OCVD1 and OCVD2 was 94 and 92%, respectively (19). In comparison with other significant OCV coverage surveys that were previously conducted in Bangladesh, the OCV coverage in the FDMN population was higher (20). However, the coverage rate was found to be comparable to the previous OCV campaigns conducted during humanitarian crises in South Sudan and Iraq (18, 21). Vaccination coverage did not vary according to sex which is similar to a study conducted in Mozambique (22). However, there were differences in coverage based on age. The lower vaccine coverage was observed among age groups equal to or >15 years in both the registered camp and the host community, which is consistent with the previous study conducted in Haiti (23).

The vaccination cards from the OCV campaign did not consistently reflect the accurate two-dose vaccination status for all respondents. Therefore, improving these aspects can contribute to more reliable and accurate documentation of vaccination status among recipients. A previous study conducted in Haiti reported that the practice of keeping vaccination cards can play a crucial role in accurately documenting vaccination status, especially in ensuring the completion of the second dose of vaccination (23).

After stratifying the coverage, we found a coverage gap between the host community with makeshift and registered camps. Considering multiple responses, the primary reason behind less OCV coverage in the host community was that they did not know about the vaccination program (41.5%); in the makeshift camp, they cited that they were busy (31.6%); and in the registered camp, they mentioned that they were unaware about vaccination (17.3%). These findings align with a previous study conducted in Bangladesh, where the most common reasons for not being vaccinated included a lack of awareness, illness, and travel commitments (19). Specifically, a lack of awareness was identified as the leading cause of decreased coverage for the MR vaccine (19). Therefore, providing clear and comprehensive messaging regarding the vaccination campaign, including information about the location, dates, and timings of the camp, can greatly contribute to improving vaccination coverage.

Perceptions and willingness to receive the vaccine differed between the host community and the camp populations. In the host community, there was a perception that they did not require the OCV vaccine, as well as no campaign was held, whereas people in camp assumed they were vulnerable and marginalized and were aware the OCV campaign was held, so the proportion was higher compared to the host community.

Another thing is that the presence of a greater population was made possible by the camp's small size, and the administration's presence ensured the efficient administration of law enforcement and other administrative tasks. As a result, the camp region had a sizable population, making it the perfect location for organizing an effective vaccination campaign.

In humanitarian settings, our study will contribute to the existing literature on cholera prevention and control. The results will offer perceptions of OCV coverage among populations that have been forcefully displaced and host communities, which will assist in informing vaccination campaigns in different humanitarian contexts.

### Limitations

Our survey has several limitations. First, clusters within the camp make it difficult to perform effective sampling because household lists are not available in registered and temporary camps as well as in the host town. Second, the participant's age was determined verbally and did not have any legal birth identification certificate, which could have caused a bias in categorizing them according to the target age group. Third, the rainy season (storms, hot, and muggy weather), especially in informal, registered camps, was troublesome as it made the work of interviewers hard to access the host community and required a boat to get to people. It was risky to travel and contact the study participants, and completed surveys could be impacted by heavy rain. Furthermore, the field study was conducted only for 15 days, which was a short duration. If the study was conducted for a longer duration, it would be possible to cover more participants from both the host community and the camp's inhabitants. Finally, our study used a cross-sectional design, which was carried out in a particular area in some of the FDMN, and may not be generalizable to the entire population.

### Recommendations

Based on our findings, we made some recommendations to increase OCV coverage in a host community as well as FDMNs in the refugee camp. First, the OCV coverage in the makeshift and registered camps was high, and therefore it is recommended to conduct the OCV campaign every alternate year. Second, because the OCV coverage in the host community was low and the risk for cholera is high as cholera is endemic in Bangladesh and the host community, the health authorities should conduct a vaccine campaign to address the reasons why people in the host community refused to get vaccinated (i.e., not knowing the place or time of vaccination).

Third, while the current OCV campaign has achieved high coverage in the camps, it is crucial to continue monitoring and evaluating its effectiveness and continue to conduct awareness campaigns, specifically targeting the host community, to educate them about the importance of OCV and the vaccination campaign.

## Conclusion

The total OCV overage was higher in the FDMN and lower in the host community. The low coverage in the host community was due to a lack of awareness of the vaccination program, a lack of knowledge of vaccination centers, and the unavailability of vaccines. During the vaccination period, conducting frequent campaigns and raising social awareness can help to enhance vaccine coverage. Furthermore, understanding the gaps in vaccination coverage and the causes of non-vaccination can be used to develop targeted interventions and strategies to improve immunization rates. In order to address comparable humanitarian catastrophes around the world, public health experts and politicians can benefit greatly from understanding and following the findings of our study.

## Data availability statement

The original contributions presented in the study are included in the article/supplementary material, further inquiries can be directed to the corresponding author.

## Ethics statement

The studies involving human participants were reviewed and approved by Institutional Review Board (IRB) of IEDCR (Institute of Epidemiology, Disease Control, and Research). Written informed consent to participate in this study was provided by the participants' legal guardian/next of kin.

## Author contributions

MQ conceived, coordinated, and designed the experiments and analyzed and drafted the manuscript. MB and AA conceived and designed the experiments and reviewed the manuscript. MS coordinated and designed the experiments. MK coordinated and drafted the manuscript. MSU coordinated the experiments and reviewed the manuscript. MN analyzed the data and drafted and revised the original manuscript. AH analyzed and drafted the manuscript. TS conceived, designed the experiments, and reviewed the manuscript. MF conceived, coordinated, designed the experiments, and analyzed and reviewed the manuscript. All authors contributed to the article and approved the submitted version.
